# The Olfactory Receptor OR51E1 Is Present along the Gastrointestinal Tract of Pigs, Co-Localizes with Enteroendocrine Cells and Is Modulated by Intestinal Microbiota

**DOI:** 10.1371/journal.pone.0129501

**Published:** 2015-06-15

**Authors:** Davide Priori, Michela Colombo, Paolo Clavenzani, Alfons J. M. Jansman, Jean-Paul Lallès, Paolo Trevisi, Paolo Bosi

**Affiliations:** 1 DISTAL, University of Bologna, Bologna, Italy; 2 Department of Veterinary Sciences, University of Bologna, Ozzano nell’Emilia (BO), Italy; 3 Wageningen UR Livestock Research, Wageningen, The Netherlands; 4 INRA, UR1341 ADNC, Saint-Gilles, France; Université de Montréal, CANADA

## Abstract

The relevance of the butyrate-sensing olfactory receptor OR51E1 for gastrointestinal (GIT) functioning has not been considered so far. We investigated in young pigs the distribution of OR51E1 along the GIT, its relation with some endocrine markers, its variation with age and after interventions affecting the gut environment and intestinal microbiota. Immuno-reactive cells for OR51E1 and chromogranin A (CgA) were counted in cardial (CA), fundic (FU), pyloric (PL) duodenal (DU), jejunal (JE), ileal (IL), cecal (CE), colonic (CO) and rectal (RE) mucosae. OR51E1 co-localization with serotonin (5HT) and peptide YY (PYY) were evaluated in PL and CO respectively. FU and PL tissues were also sampled from 84 piglets reared from sows receiving either or not oral antibiotics (amoxicillin) around parturition, and sacrificed at days 14, 21, 28 (weaning) and 42 of age. JE samples were also obtained from 12 caesarean-derived piglets that were orally associated with simple (SA) or complex (CA) microbiota in the postnatal phase, and of which on days 26–37 of age jejunal loops were perfused for 8 h with enterotoxigenic *Escherichia coli* F4 (ETEC), *Lactobacillus amylovorus* or saline (CTRL). Tissue densities of OR51E1+ cells were in decreasing order: PL=DU>FU=CA>JE=IL=CE=CO=RE. OR51E1+ cells showed an enteroendocrine nature containing gastrointestinal hormones such as PYY or 5HT. *OR51E1* gene expression in PL and FU increased during and after the suckling period (p<0.05). It was marginally reduced in offspring from antibiotic-treated sows (tendency, p=0.073), vs. control. Jejunal *OR51E1* gene expression was reduced in piglets early associated with SA, compared with CA, and in ETEC-perfused loops vs. CTRL (p<0.01). Our results indicate that *OR51E1* is related to GIT enteroendocrine activity. Moreover age, pathogen challenge and dietary manipulations influencing the gastrointestinal luminal microenvironment significantly affect the *OR51E1* gene expression in GIT tissues presumably in association with the release of microbial metabolites.

## Introduction

The superfamily of olfactory receptors (ORs) is formed by a very large number of G-protein coupled receptor proteins that detect volatile odorant molecules. They were initially discovered in the olfactory epithelium, but recent evidence shows that several of them are well expressed in the respiratory tract and in other tissues [1], where their function is still unclear. In the gastrointestinal tract (GIT), they are detected in enterochromaffin cells and can affect the secretion of serotonin (5-hydroxytryptamine; 5HT) in response to fragrant molecules [2], with subsequent effects on gut motility [3]. During a comparative investigation of the transcriptome between the gastric fundic and the pyloric mucosae of pigs [4], it was evidenced that one gene- *OR51E1* (olfactory receptor, family 51, subfamily E, member 1, previously named GPR164)—was expressed more than other genes assigned to this category.

Deorphanization studies by cell-reporter systems evidenced that 3- and 4- methyl-valeric acids [5], nonanoic acid [6] and butyric acid [7] are agonists of this receptor. The sensitivity of OR51E1 to butyrate is of particular interest due to the multiple implications of this enteric bacterial metabolite in regulating GIT tissues in normal and pathological states [8].

Butyrate-sensing olfactory receptor OR51E1 has been identified only in GIT carcinomas [9], but its relevance for GIT physiology has not been considered so far.

In mammals, the maternal environment is a major determinant shaping the gut microbiota in early life [10,11]. Initial qualitative and quantitative colonization by environmental bacteria in the neonate and in the suckling mammal may play a role in the programmed maturation of the GIT. Both transiting and colonizing microbiota may contribute to the latter [12,13]. The effects could be exerted via multiple ways: influencing nutrient availability, synthesis of gut-active metabolites, interactions with host systems for detection of xenobiotics, involvement in the induction and activation of innate and acquired immune defences, via the differentiation and proliferation of GIT cells and development of the architecture of the intestinal mucosae. A part of these actions is explained by the intestinal release of the peptide YY (PYY) stimulated by luminal butyrate [14].

Microbial imprinting has been studied mostly in mice, and more frequently for the maturation of the immune system, and in poultry in a few studies [15,16]. The relevance of the maternal environment is also indirectly confirmed by the copious literature promoting breastfeeding in babies. Both inadequate early contact with favourable bacteria and their insufficient seeding are also implicated in the ontogeny of several metabolic and immune-related diseases in humans [17].

Here, we investigated the OR51E1 distribution along the GIT and its cellular association with some relevant endocrine markers, e.g. 5HT and PYY in young pigs. Furthermore, we hypothesized that OR51E1 tissue distribution may vary with changes in GIT microbiota or by microbial challenge. For investigating the former, we used piglets born from sows either or not treated with antibiotics during the gestation and lactation phase and piglets born by caesarean section and orally associated with different microbiota in the postnatal phase.

## Materials and Methods

### Study A: distribution of OR51E1 along the GIT

Ethics statement: The procedures carried out on the pigs were conducted in compliance with Italian laws on experimental animals and were approved by the Ethic-Scientific Committee for Experiments on Animals of the University of Bologna (Protocol submission to Italian Ministry of Health, number 34613-X/10).

Three Large White littermate pigs, one female and two males, were fed a typical post-weaning diet and housed individually in pens with a mesh floor in a temperature-controlled room and tap water freely available. At six weeks of age and a body weight (BW) of 13.4 ± 0.4 kg, the piglets were slaughtered and whole tissue samples for each of the following parts of the GIT were collected for immunohistochemistry analysis: gastric cardia (CA), fundus (FU), pylorus (PL); duodenum (DU), jejunum (JE), ileum (IL); cecum (CE), colon (CO) and rectum (RE).

### Study B: *OR51E1* gastric gene expression in pigs born to sows either treated orally with an antibiotic around parturition or not

Ethics statement: The experiment was designed and executed in 2010 in compliance with French and European law (Decree No. 2001–464 29/05/01, 86/609/CEE) for the care and use of laboratory animals. At that time (2010) getting approval from an ethic committee was not mandatory. One of us (JPL) held the authorization certificate No. 006708 for experimentation on living animals delivered by the French Veterinary Services. INRA Saint-Gilles, including the on-site slaughterhouse has an institutional license (agreement No. A35-622) from the French Veterinary Services.

The study aimed at assessing if early disturbances in microbial colonization have an impact on *OR51E1* gene expression in the stomach. This was the part of the GIT showing in the highest number of OR51E1 staining cells along its different segments, in study A. Stomach tissue samples were harvested from offspring born to control mothers (CTRL, n = 12) or mothers treated with the antibiotic amoxicillin around parturition (day -10 till day + 21) (ATBC, n = 11) in a larger study described in more detail by Arnal et al. [18, 19]. Broad spectrum antibiotic amoxicillin (Vetrimoxin PO containing 10% amoxicillin; CEVA Santé Animale, Loudéac, France) was provided daily to the sows (40 mg/kg BW) orally together with their morning meal (2 kg/day). They were fed the rest of their daily feed allowance without supplemental antibiotic. Amoxicillin was used as antibiotic because the experiment with pigs was primarily set up as a model for humans in the context of so called “DOHaD” (developmental origins of health and disease). Two experiments reporting long-term effects of neonatal antibiotic administration on gut physiology (barrier) had already been published in rodents using amoxicillin [20,21].

Litter size was adjusted within treatment groups at n = 12 piglets per litter at the end of farrowing. Four pigs per sow were randomly assigned to slaughter at the ages of 14, 21, 28 (age of weaning), and 42 days, respectively. Piglets were selected from all available litters to keep the mean BW and its variation in the sub-groups to be sacrificed as equal as possible. However, for two litters per each sow treatment, low numbers of offspring (but equalized piglet numbers during the suckling period, with fosters) did not allow slaughtering one pig per litter each time (given the fact that the general plan included also four additional pigs for the long-term part of the experiment, as already published [18,19]). Thus four pigs less were sampled for the slaughtering at 28 d and 42 d, and total eighty four offspring pigs are included in the present data set. This experimental design allowed testing the effect of treatment (ATBC vs. CTRL) within litters.

Sows and piglets were fed balanced diets formulated to cover all known nutritional requirements for gestating and lactating sows, and for starting (pre-starter and starter), respectively. Complete feed composition is available from Arnal et al. [18]. Sows were fed the gestating diet (3.5 kg/day) or the lactating diet (*ad libitum*) in two meals. Offspring had *ad libitum* access to their successive diets. At slaughter, the whole tissue samples of the stomach were obtained from FU and PL mucosae of each pig, and were immediately snap-frozen in liquid nitrogen for molecular analysis at a later stage.

### Study C: Effects of early microbial association and intestinal loop treatments in caesarean-derived (CD) piglets

Ethics statement: The protocol of the study was approved by the Committee on the Ethics of Animal Experiments of Wageningen University and Research Centre in Lelystad, The Netherlands (Permission Number: 2012083.e).

Twelve piglets from sows [(Great York × Pie) × ‘Dalland’ cross] were obtained by caesarean delivery (day 0) and were divided over two microbiota association treatments housed in separate clean, non-sterile rooms and balanced for BW and litter of origin. Piglets were housed in two pens per room suited with an automatic feeding system for supply of a moist diet. Average birth weight was 1273 ± 138 g and 1275 ± 153 g for the two treatment groups, respectively. Each time at 1 h and 5 h after birth, each CD-piglet received 45 mL pasteurized (30 min at 60°C) sow colostrum by oral gavage [22,23]. All piglets received a simplified starter microbiota consisting *of Lactobacillus amylovorus* (LAM) (3.6 × 10^7^ CFU), *Clostridium glycolicum* (5.7 × 10^7^ CFU), and *Parabacteroides* spp. (4.8 × 10^7^ CFU) by oral inoculation (2 mL) on days 1, 2, and 3 after birth. These bacterial species are, among the phylogenetic groups, the most frequently identified in the intestine of piglets and were proposed by Laycock et al. [24] as intestinal colonization microbiota for gnotobiotic pigs. On days 3 and 4 of age, the piglets received either a complex microbiota by providing them with 2 mL of an inoculant consisting of 10% saline diluted feces of an adult sow (complex association, CA) or a placebo inoculant (10% saline) (simple association, SA). Piglets were fed *ad libitum* a milk replacer diet during a period of 5 d (days 0–4). It consisted of bovine skimmed milk powder, whey powder, vegetable oil, hydrolysed wheat protein, wheat starch, sucrose and a vitamin and mineral premix, and contained 230 g crude protein per kg milk replacer. A moist diet based on whey powder, maize, wheat, toasted full fat soybeans, oat flakes, sucrose, soybean meal, vegetable oil, coconut oil, wheat gluten, potato protein, rice protein, and brewer’s yeast was fed during the remainder of the study.

On days 26–37 of age, intestinal tissue samples were obtained from intestinal loops prepared for applying the “*in vivo* small intestinal segment perfusion” (SISP) technique, as described by Nabuurs et al. [25,26]. In brief, the abdomen was opened along the *linea alba* and three small intestinal segments of 20 cm in length each were made in the mid-jejunum, starting at a distance of 200 cm distal to the ligament of Treitz. These segments retained their vascularization and were cannulated with a rubber tube at the proximal and distal ends to perfuse and collect fluid, respectively.

Three different isolated jejunal loops per pig were perfused with 8 mL fluid for 8h (saline with 0.1% glucose and 0.1% amino acids per h) containing the following solutions: enterotoxigenic *Escherichia coli* F4 (ETEC) as pathogenic strain (5.5 x 10^9^ CFU per loop), or LAM as beneficial strain (7.5 x 10^9^ CFU per loop) or saline as control (CTRL). At the end of the SISP study, a sample of jejunal tissue per loop was collected and snap-frozen in liquid nitrogen for further molecular analysis.

#### Immunohistochemistry

Gastrointestinal samples were washed with PBS and fixed in 4% buffered formalin overnight. Standard procedure for paraffin embedding was followed and then 8 μm-thick of cross-sectional sections of the organ tissue were obtained from microtome cutting and mounted on microscope poly-L-lysine coated slides (Sigma-Aldrich, Milan, Italy). For immunohistochemical analysis, the sections were dewaxed, rehydrated and heated in citrate buffer pH 6 for 10 min at 700 w in microwave for antigen retrieval. Then, they were pre-incubated in PBS + 0.3% TritonX + 5% of donkey or goat normal serum for 1 h and incubated with the following primary antisera diluted in PBS + 5% serum overnight at +4°C: rabbit anti-human OR51E1 (code GTX100361; GeneTex Prodotti Gianni, Milano, Italy) 1:200, mouse anti- CgA (code MON 9014, Monosan Xtra, DBA Italia, Segrate, Italy) 1:600, mouse anti-5HT (code Ab16007, Abcam, Cambridge, UK) 1:200, guinea pig anti-pig PYY (code PAB17185; ABNOVA, Taipei, Taiwan) 1:1000. The porcine epitope for OR51E1 antibody shares 96% of homology compared to humans by sequence blast while the anti-GgA antibody was previously successfully tested on porcine CgA [27]. Then, the tissue sections were incubated with the following secondary antibodies for 1 h at room temperature: donkey anti-rabbit Alexa 488 (code ab150073; Abcam) 1:500, donkey anti-mouse Alexa 594 (code A21203; Molecular Probes, Eugene, OR, USA) 1:600, goat anti-guinea pig TRITC (Jackson ImmunoResearch Laboratories, Inc., West Grove, PA, USA) 1:100. The slides were finally mounted via VECTASHIELD Mounting Medium with DAPI (Vector Laboratories, Inc., Burlingame, CA, USA) for microscope visualization. Preparations were examined on a Nikon Eclipse Ni microscope (Nikon Instruments, Sesto Fiorentino, Italy) equipped with the appropriate filter cubes to distinguish the fluorochromes employed. The images were recorded with a Nikon DS-Qi1Nc digital camera (Nikon Instruments) and NIS software (BR 4.20.01; Nikon Instruments).

In order to analyse a large and comparable area for each slide, the mosaic software procedure was used to capture a large composite microscope image, and only the mucosal area correctly cut transversally were selected as regions of interest for each slide. Three slides per sample and a minimum of 3 mm² per region of interest in each slide were analysed. Immunoreactive cells were visually evaluated for the reactivity and manually counted.

#### RNA Isolation and gene quantification by real-time RT-qPCR

Total RNA was isolated from the intestinal tissue samples collected in trials B and C according to FastPure RNA kit (TaKaRa Bio Inc., Shiga, Japan). All other procedures were in agreement with the manufacturer’s protocol. RNA purity and integrity were evaluated by Agilent Bioanalyzer 2100 (Agilent Technologies, Palo Alto, CA) just before real-time quantitative PCR (RT-qPCR) analysis.

The expression of *OR51E1* gene was quantified by RT-qPCR. One microgram of total RNA was reverse-transcribed using the ImProm-II Reverse Transcription System (Promega, Milan, Italy). Primers were designed based on a specific porcine nucleic acid sequence (Gen-Bank database) using Primer 3 version 0.4.0 (http://frodo.wi.mit.edu/primer3/). The RT-qPCR reactions were performed in a LightCycler Real-Time PCR Systems (Roche Applied Science, Bazel, Switzerland) by a shuttle PCR (2 steps) following the procedure described by Trevisi et al. [28]. The expression of data was normalized by geometric mean of expression data for two housekeeping genes: hydroxymethylbilane synthase (*HMBS*) and ribosomal protein L4 (*RPL4*) genes for gastric tissue samples (study B), and *HMBS* and TATA box binding protein (*TBP*) genes for jejunal tissue samples (study C), following Nygard et al. [29]. Primers and amplification conditions for *OR51E1* and housekeeping genes are reported in Table 1.

**Table 1:** Primers information and RT-qPCR conditions used in the trials.

Gene^a^	NCBI accession number	Oligo sequence (5'→3'): Forward—Reverse	Amplicon length	Annealing temperature
***OR51E1***	XM_005656617.1	CGCGTCAACATCATCTATGGC CGCACACATGGGAGACACAC	160pb	59°C
***HMBS***	DQ845174	AGGATGGGCAACTCTACCTG GATGGTGGCCTGCATAGTCT	83 bp	62°C
***RPL4***	DQ845176	CAAGAGTAACTACAACCTTC GAACTCTACGATGAATCTTC	122 bp	60°C
***TBP I***	DQ845178	AACAGTTCAGTAGTTATGAGCCAGA AGATGTTCTCAAACGCTTCG	153 bp	60°C

^a^ *OR51E1*, olfactory receptor, family 51, subfamily E, member 1; *HMBS*, hydroxymethylbilane synthase; *RPL4*, ribosomal protein L4; *TBP*, TATA box binding protein.

#### Statistics

Statistical analysis of data (S1, S2 and S3 Tables) was carried out using the MIXED procedure of SAS (version 9.3; SAS Institute Inc., Cary, NC, USA). In study A, the effect of the point of measurement was calculated considering the repeated measures inside each pig. In study B, the effects of sow’s treatment (against an error calculated between litters), offspring age at slaughter (error within litters), point of measurement (FU and PL, error within pigs), and the relative first order interaction terms were tested. For the effect of age at slaughter, the following contrasts were calculated: “Linear effect during suckling”, inside un-weaned groups (that is: offspring aged 14, 21 and 28 days), and “suckled *vs*. weaned” (that is: un-weaned pigs aged 14, 21 and 28 days *versus* weaned piglets aged 42 days). In study C, the effects of early microbiota association (against an error calculated between pigs), of loop treatment (error within pigs), and the interaction term between early microbiota association and loop treatment were tested. For the loop experiment, treatments were contrasted against control infusion. Results are presented as least-squares means and pooled SEM. Least-squares means comparisons for each interaction were made only when a tendency (p≤0.10) for an interaction between these terms was observed. Effects were considered significant at p≤0.05 and as a trend at p≤0.10.

## Results

### Trial A

**Fig 1:**
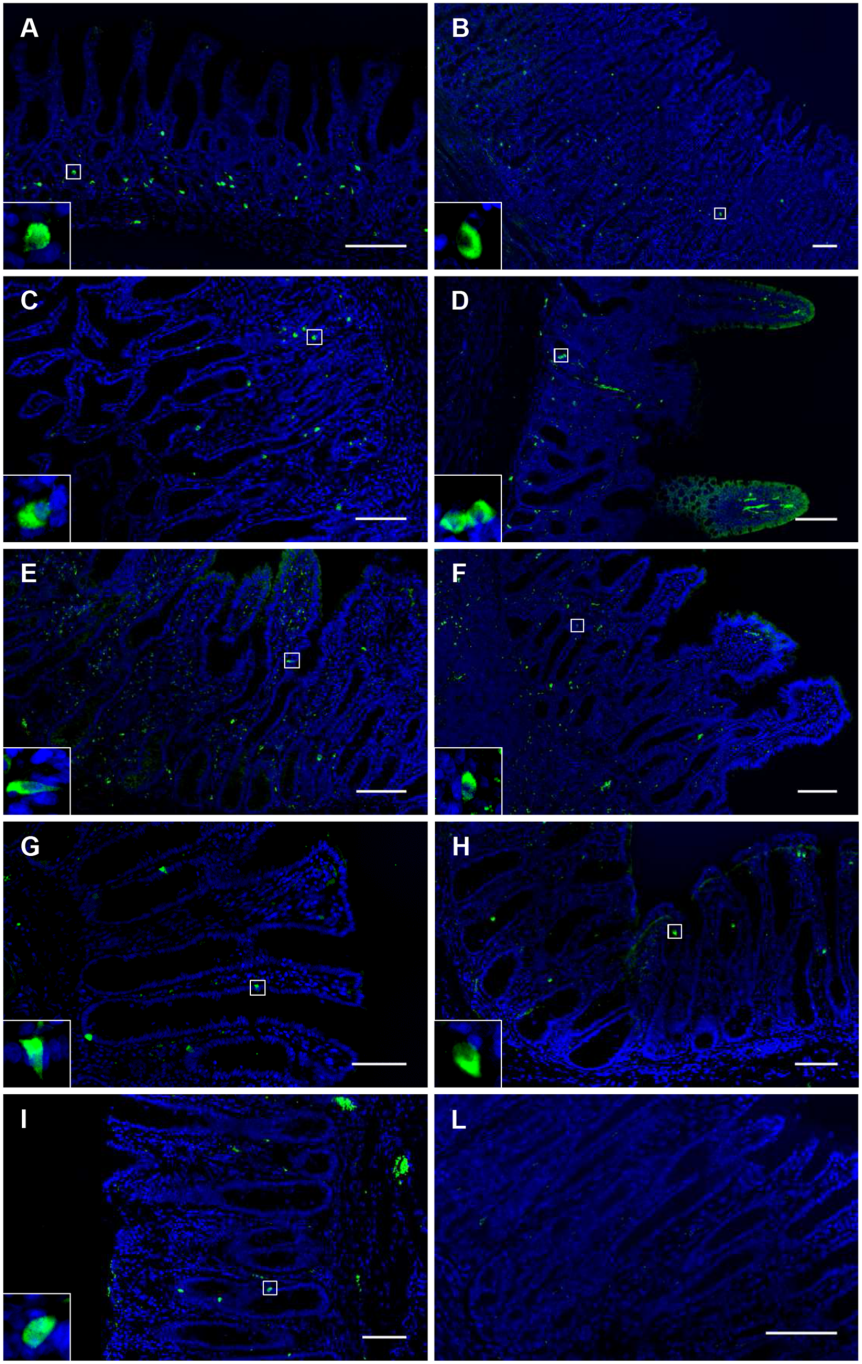
Visualization of OR51E1 tissue distribution in the gastrointestinal tract. A = cardia, B = fundus, C = pylorus, D = duodenum, E = jejunum, F = ileum, G = cecum, H = colon, I = rectum, L = pylorus, control (without primary antibody). Scale bar = 100 μm. The OR51E1 immunostaining distribution is mostly in the bottom half of the mucosa in each tissue. There is a higher density of OR51E1+cells found in the gastric mucosa (A–C) with a peak density in the pylorus (C). The morphology of the OR51E1+ cells, as magnified in a small square on each picture is generally of the close-type with a round shape but sometimes they show an open-type morphology with a triangular shape (e.g. Fig 1, E, G, H), particularly in the top half of the mucosa, closer to the lumen.

**Fig 2:**
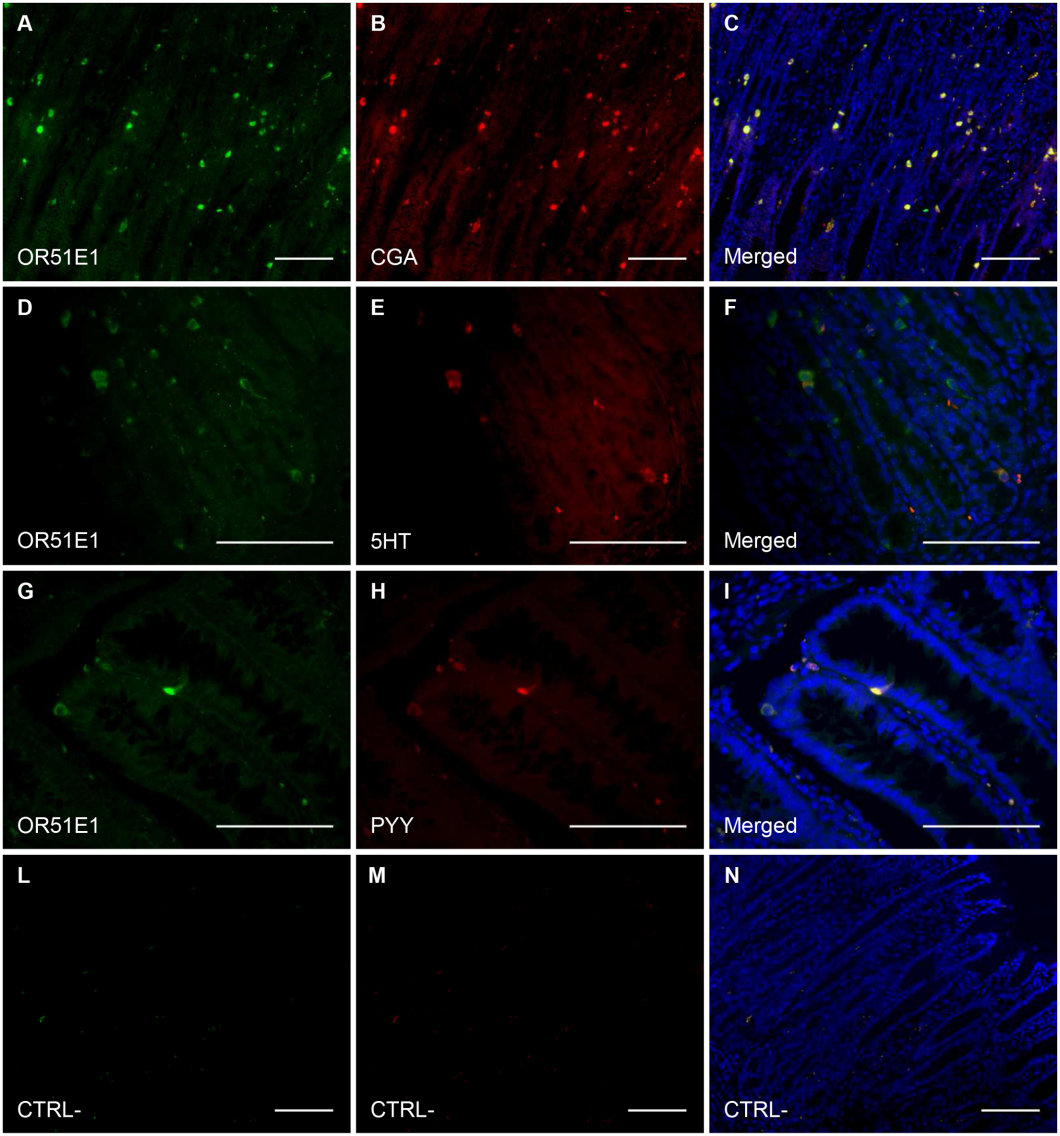
Visualization of OR51E1 co-localization with enteroendocrine cells and some hormones. A–F = pylorus, G-I = colon, L–N = pylorus, control (without primary antibodies). Scale bars = 100 μm. Almost all the OR51E1+ cells have an overlapping staining with the cells positive for the enteroendocrine marker chromogranin A (A, B, merged in C) showing the endocrine nature of these cells. A subset of these cells also contain hormones such as serotonin (C, D, merged in E) and peptide YY (G, H, merged in I) suggesting a possible role for OR51E1 in the control of hormone release. In control images only erythrocytes showed an auto-fluorescence.

The immunohistochemical visualizations for *OR51E1* staining in each tissue and for co-localizations with CgA, 5HT and PYY are presented in Figs 1 and 2, respectively.

**Fig 3:**
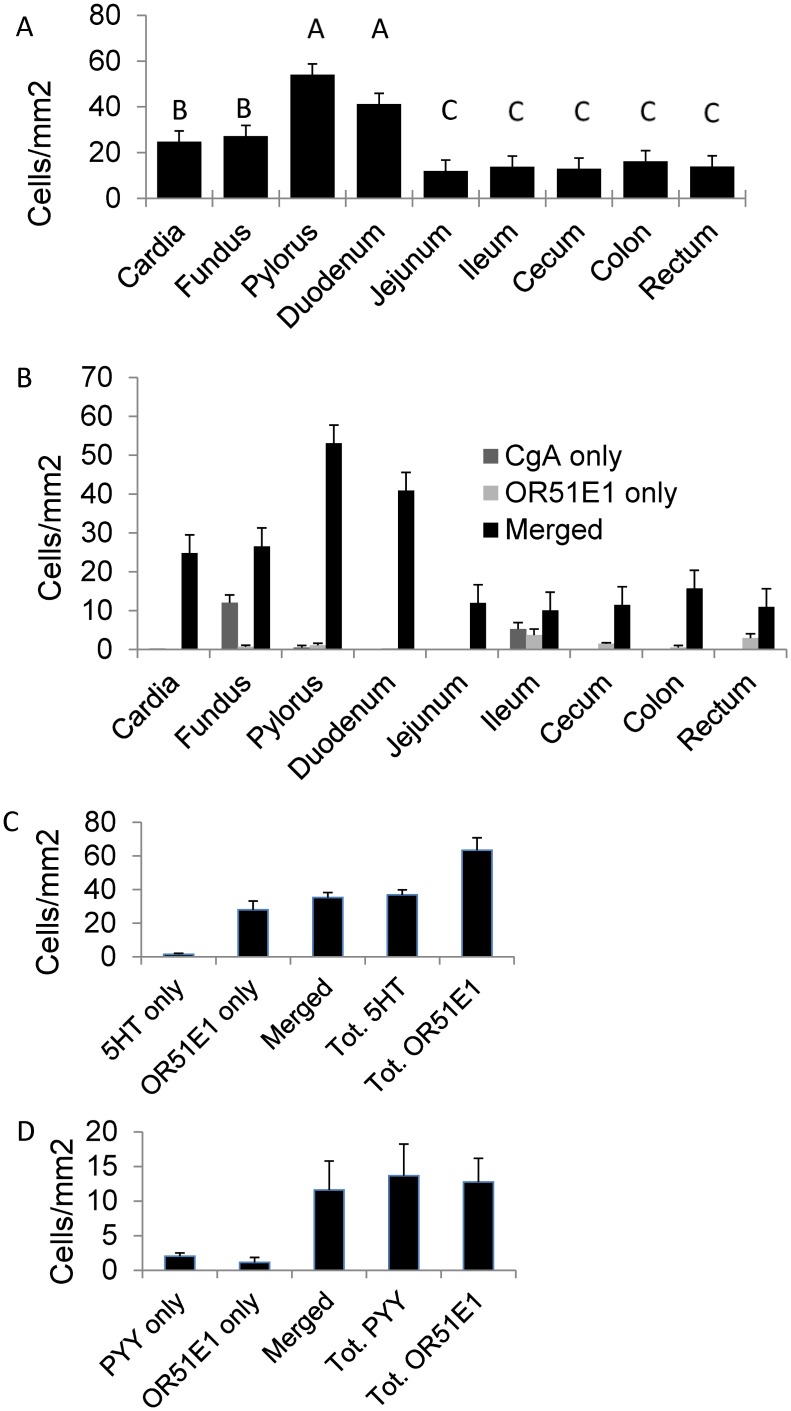
Immunostainings for OR51E1, chromogranin A (CgA), serotonin (5HT) and peptide YY (PYY) in different mucosae of the GIT. Counts (with standard errors of means) of: A) OR51E+ cells. PL = DU>CA = FU> JE = IL = CE = CO = RE (A,B,C: p<0.01); B) cells co-staining (= Merged) or not, for OR51E1 and CgA; C) cells co-staining (= Merged) or not, for OR51E1 and 5HT in jejunum; D) cells co-staining (= Merged) or not, for OR51E1 and PYY in colon.

All tissues samples from the various segments of GIT displayed cells staining for OR51E1. Tissue densities of OR51E1+ cells were in the decreasing order: PL = DU>CA = FU> JE = IL = CE = CO = RE (p<0.01) (Fig 3A). OR51E1+ cells stained also for CGA, with co-staining varying from 100% (CA, JE) to 73% (IL) (Fig 3B). In PL and CO, 56% and 91% OR51E1+ cells co-stained with 5HT and PYY, respectively. Conversely, 95% 5HT+ and 85% PYY+ cells co-stained with OR51E1 (Fig 3C and 3D).

### Trial B

**Fig 4:**
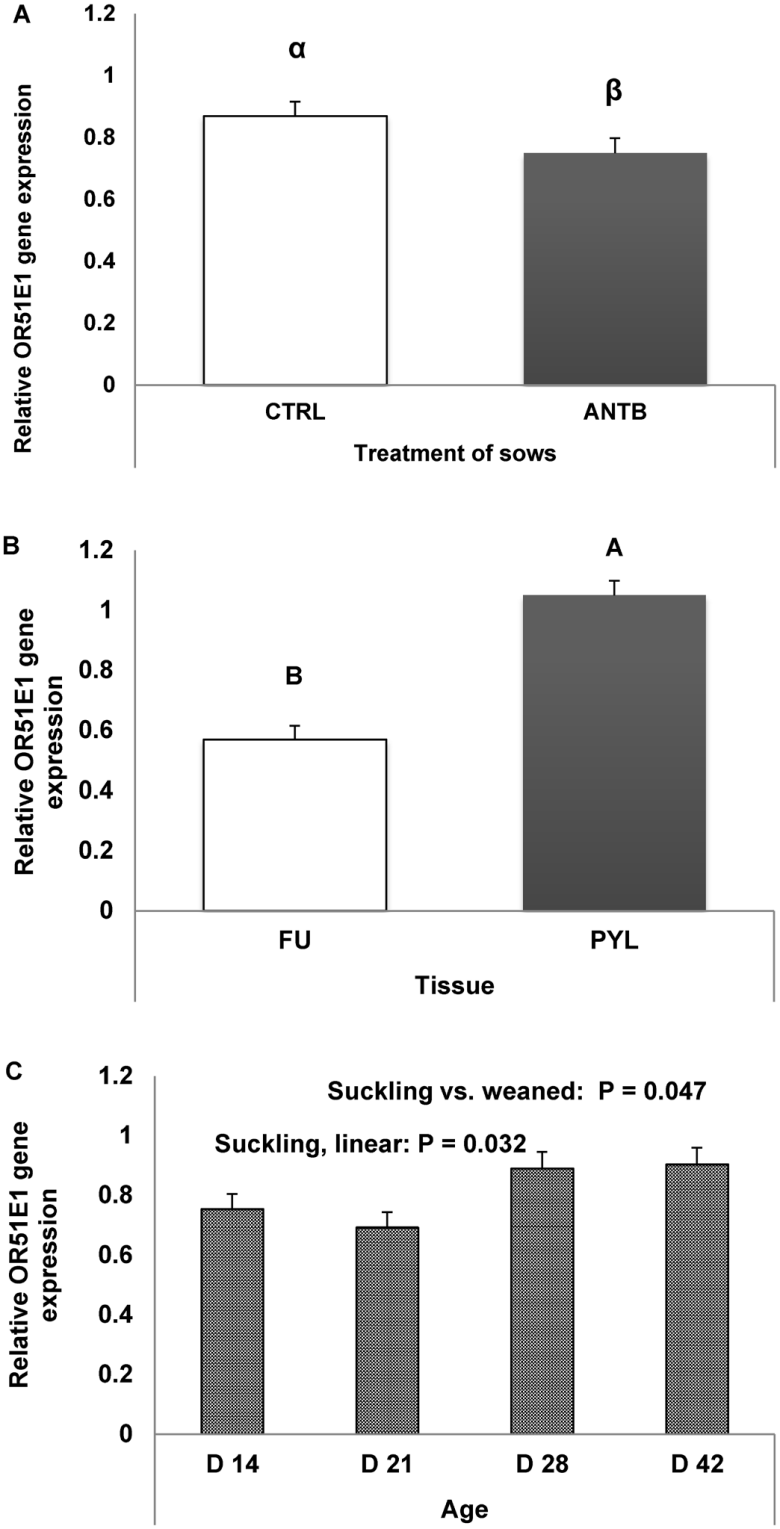
Effects of antibiotic treatment of sows in gestation—lactation (A), offspring gastric tissue location (FU = fundic; PL = pyloric) (B), and offspring age (weaning at 28 days) (C), on relative gene expression of *OR51E1* in the stomach. Bars represent standard errors. No statistically significant interaction between the factors was seen. Orthogonal contrast analyses were conducted as follows: 4A: control vs. Antibiotic treatment (all ages and gastric sites considered) (α;β: p = 0.073); 4B: Fundic vs. Pyloric region (all sows’ treatments (CTL and ANTB) and age at slaughter considered) (A,B: p = 0.005); and 4C: Suckling (day 18 + day 21 + day 28) vs. weaned (day 42) period (all sows’ treatments (CTL and ANTB), age at slaughter and gastric site considered).

The results showed that OR51E1 gene expression in small intestinal tissue differed about similarly for intestinal loops imposed to the various perfusion treatments in both microbiota association groups of pigs, as confirmed by the lack of significant interactions between these factors. Therefore, the effects of these factors are presented independently. The antibiotic treatment to the sows in the end gestation—lactation phase marginally reduced *OR51E1* gene expression in offspring gastric PL and FU regions (tendency, p = 0.073) (Fig 4A) *vs*. control sows. Moreover, *OR51E1* gene expression was higher in PL than in FU (p = 0.005) (Fig 4B).

*OR51E1* gene expression in PL and FU increased during the suckling phase (p = 0.032), that was covered by samplings at d18, d21 and d28, and was higher in the weaned pigs (sampled at d42) compared with the whole pre-weaning period (p = 0.047) (Fig 3C).

### Trial C

**Fig 5:**
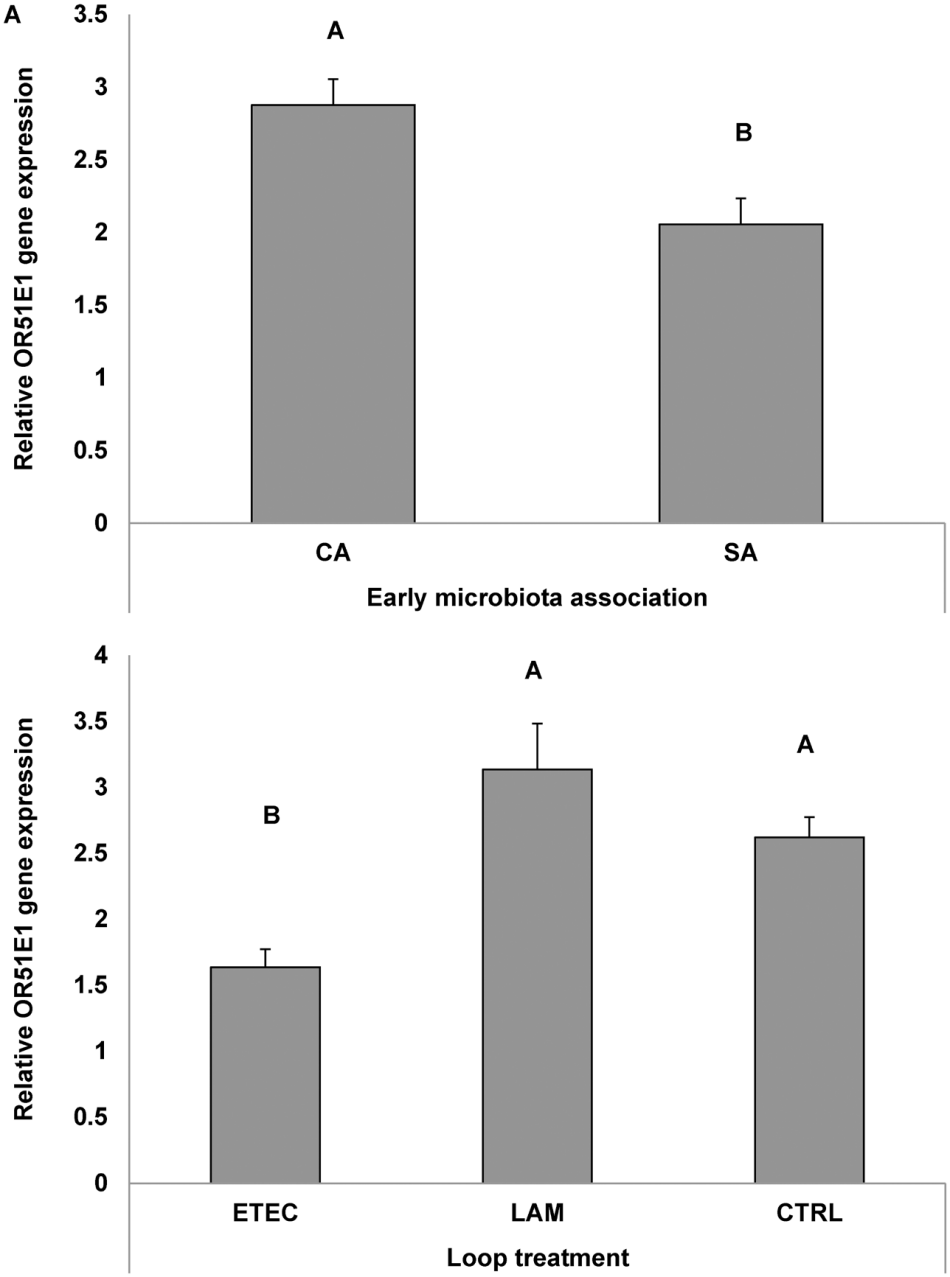
Effects of early association with simple (SA) or complex microbiota (CA) to piglets in the post natal phase, and of intestinal loop treatment (pre-perfusion with saline, *Lactobacillus amylovorus* or ETEC) at 4 to 5 weeks of age on the relative gene expression of *OR51E1*, after 8 h of loop perfusion. Bars represent standard errors. No statistically significant interaction between the factors was observed. 4A) Effect of early microbiota association (A, B: p = 0.003); 4B) Effect of loop pre-perfusion treatment; statistical significance is against saline treatment (A, B: p<0.001), ETEC = enterotoxigenic *Escherichia coli* K88; LAM = *Lactobacillus amylovorus*; CTRL = saline.

The results showed that *OR51E1* gene expression in small intestinal tissue differed about similarly in both microbiota association groups of pigs and in intestinal loops imposed to the various perfusion treatments within each pig, as confirmed by the lack of significant interactions between these factors. *OR51E1* gene expression was lower in piglets early associated with SA compared with CA (p = 0.003, Fig 5A), and in ETEC-perfused jejunal loops *vs*. CTRL (p<0.001, Fig 5B). LAM perfusion had no effect on *OR51E1* gene expression.

## Discussion

Olfactory receptors belong to the largest G protein-coupled receptor family in mammals and are generally thought to be expressed in the olfactory epithelium, to detect volatile odorants [30–33]. However, recent studies reported the presence of ORs in non-olfactory tissues, where their roles remain unclear [34,35]. Our previous transcriptomic study [4] indicated the abundance of *OR51E1* mRNA in the gastric mucosa of the pig, an observation never reported for this tissue before. Inside this investigation, exploring the differential gene expression between oxyntic and pyloric tissues by microarray, we found that *OR51E1* gene expression was similar to the expression of one of the two gastric housekeeping genes (*HMBS*) and also 3.5 times higher than the expression of the second higher expressed OR gene (*OR4X1*) (unpublished data).

We hypothesize that OR51E1 could have a chemosensing role in the detection of volatile substances along the GIT, could be involved in some important endocrine functions, and finally may be modulated by several factors related to the gut environment including the residing microbiota and challenge by pathogenic bacteria.

The protein expression of *OR51E1* was detected all along the GIT (Fig 3A), from the gastric cardia to the rectum, and this wide distribution suggests an important, still undiscovered role for this receptor. The highest relative number of OR51E1+ cells in the pylorus is consistent with a role for the distal stomach in sensing nutrients and xenobiotics [36]. Comparing the fundic and pyloric mucosae in growing pigs, it was also evidenced that several genes related to antimicrobial peptide secretion and cytoprotection were highly expressed in the pylorus: lysozyme, polymeric immunoglobulin receptor, cytochrome P450 family 3 subfamily A, polypeptide 46, secretoglobin, family 1A, member 1 (uteroglobin) [4]. The pyloric region is an interface between different micro environments in the stomach and in the proximal small intestine. Our results show that OR51E1 co-localizes with the enteroendocrine cell marker CgA all along the GIT for a percentage varying from 78% to 100% (Fig 3B). This suggests that this olfactory receptor is constitutive to this type of cells and reveals their enteroendocrine nature. Therefore, our findings support the hypothesis that the function of OR51E1 may be related to an endocrine response, though this requires further confirmation. In addition, 96% and 85% of the enteroendocrine cell subsets containing 5HT and PYY respectively, co-localize with OR51E1 (Fig 3B and 3C). Enterochromaffin cells are widely distributed along the mucosal surface of the GIT and respond to chemical and physical signals, thus releasing 5HT to activate intrinsic and extrinsic nerves, and producing physiological responses such as GIT motility [37] and immune activation [38]. Serotonin has been shown to co-localize with olfactory receptors in solitary pulmonary neuroendocrine cells where a volatile stimulation-dependent release of 5HT is detected [39]. Conversely, in FU mucosa, co-staining between OR51E1 and CgA was not so high (69% merged cells of total CgA+ cells). In FU different enteroendocrine cell types are present, e.g. D and P/D1 cells secreting somatostatin and ghrelin, respectively, and enterochromaffin-like cells, secreting histamine.

The second tissue with high density of OR51E1+ cells revealed to be the duodenum. Also for this location there was an almost full overlap (99%) with CgA staining. Duodenal enteroendocrine cells are primarily sources of gastric inhibitory peptide (GIP, secreted by K cells) [40] and cholecystokinin (CKK, secreted by I cells) [36]. Furthermore, these cells were shown to be equipped with several receptors linked to sweet and bitter tastes [36]. They also displayed the necessary proteins (α-transducin and α-gusducin) to translate this signal into the endocrine secretion. Collectively, these observations may suggest a possible link between *OR51E1* expression and modulation of endocrine activity.

Bacterial metabolites as 3- and 4- methyl-valeric acids, nonanoic acid and butyrate were recently proposed to be activators of OR51E1 [5, 6, 7]. It is already established that the sensing of bacterial metabolites participates to the local hormonal control of the gut function. For example, propionate has been shown to cause the release of PYY and to slow down intestinal transit [41]. Our observation that colonic PYY+ cells harbored also OR51E1 adds to what has been seen for the two fatty acid receptors GPR43 and GPR41 in human colon [42,43]. However, OR51E1 receptor was more densely located in the gastric than in the intestinal mucosae (Fig 1, Fig 3B). In gastric digesta of young pigs, butyrate was detected at different concentrations largely depending on dietary condition [44–46] which may have affected the presence of butyrate-producing bacteria. In the stomach of suckling pigs, lactate-utilizing butyrate-producing bacteria may take benefit of the lactate producers growing on lactose from milk or of the cross-feeding of partial breakdown products from other substrates, as proposed for human gut [47]. Furthermore, butyrate-producing Clostridia isolated from pigs can use mucins for growth [48].

With trial B and C we provide evidence that several experimental factors modulate *OR51E1* gene expression along the gut. This led to variations in the numbers of OR51E1-expressing cells and/or in the transcript level in the expressing cells. Feedback mechanisms, differential activation of transcription factors as well as epigenetic regulation have been evidenced for *OR51E1* gene expression in olfactory bulbs [3]. Several circulating hormones, mostly related to the control of food intake and energy balance can modulate olfactory epithelium [3]. Conversely, there is scarce knowledge on regulation of *OR* genes beyond the nose. Postnatal GIT colonization is favored by the multiple encounters of a large variable pool of bacteria from the environment. The progressive raise of gene expression during the suckling period and from this phase to the post-weaning period suggests a dependence of *OR51E1* gene regulation on mucosa or microbiota maturation (Fig 4C). In an earlier experiment, caesarean-derived piglets that were orally dosed with an inoculant consisting of sow’s diluted feces in the first days after birth had a more complex intestinal and fecal microbiota for the first four weeks of life, compared to those receiving only the simplified pool of bacteria (as the one used in our study) [49]. The effect of microbiota association treatment (simple or complex) on the jejunal microbiota community has been confirmed in contemporary piglets in the same study (A.J.M. Jansman et al, manuscript in preparation). Furthermore, it was evidenced that an early microbiota association treatment also influenced gastric mucosa transcriptome at two weeks of age, with down-regulation of several metabolic pathways related to cell replication and immune response, with SA treatment [50]. Early association with a complex microflora (study C) stimulated the intestine to have a high *OR51E1* gene expression (Fig 5A), and this may have been related to the higher complexity of the gastric microbiota. Furthermore, the same early imprinting from the environment can also help explain the trend for decreased *OR51E1* gene expression in piglets that were reared from sows treated with an antibiotic, as compared to those from control sows (study B) (Fig 4A). In fact, sow’s antibiotic treatment affected offspring ileal microbiota composition (approximate genus-level microbial groups) during the first weeks of life and reduced microbiota diversity on day 14 of age [18]. Unfortunately, gastric digesta was not sampled in the present study, so possible changes in microbiota composition, as induced by antibiotic treatment, could established and not be linked to changes *OR51E1* gene expression in stomach tissue.

The second goal of the study C was to investigate the effects of beneficial or harmful conditions coupled to differences in intestinal microbiota composition on *OR51E1* gene expression in the gut. The *OR51E1* gene expression was also strongly decreased in a pathogenic condition induced by ETEC challenge (Fig 5B). Previous research had demonstrated that ETEC challenge in the SISP test increased the number of ETEC attached to the small intestinal mucosa in ETEC perfused loops, causing a decrease in net fluid absorption [51]. This is certainly related to the strong damage of the tissue always observed during ETEC infection [52] and likely by the lower microbiota complexity induced by an early association treatment with a limited number of microbial species (SA). Conversely, *Lactobacillus amylovorus* did not affect *OR51E1* gene expression compared to CTRL treatment, suggesting that this supposed beneficial strain did not interfere with normal OR51E1 receptor expression. Nevertheless, the results obtained with SISP model in study C allows to hypothesize the existence of a lumen-driven regulation of *OR51E1* gene expression. Conversely, systemic-driven regulation should not be hypothesized because *OR51E1* down regulation was not observed in intestinal loops perfused with *Lactobacillus* or saline. In fact in the SISP model, all the loops share the same systemic blood supply. Thus, paracrine regulatory mechanism for *OR51E1* gene expression can be envisaged.

## Conclusions

Our results indicate that age, pathogen challenge and dietary manipulations affecting the gut luminal micro-environment and the intestinal microbiota modulate *OR51E1* gene expression in GIT tissues. The expression of this receptor seems be related particularly to the factors that affect complexity of the microbiota. Moreover, we showed that OR51E1 is a receptor often located in enteroendocrine cells all along the GIT in which this receptor could have an important role to modulate the secretion of some gastrointestinal hormones. Further investigations are needed to elucidate the functional implications of these findings and to identify which bacterial species are directly involved in *OR51E1* modulation.

## Supporting Information

S1 Table Counts of OR51E1+ cells per each subject and point of measure in study 1. Density values, n cells/mm^2^.
(DOCX)

S2 Table Individual expression of *OR51E1* and of housekeeping genes for study 2. Suckling sow number, its treatment, and the final piglet age are reported for each subject. Values are per each point of sampling and in gene copies/mg RNA.
(DOCX)

S3 Table Individual expression of *OR51E1* and of housekeeping genes for study 3. Early microbiota association treatment of the pig and intestinal loop treatment are reported for each observation. Values are in gene copies/mg RNA.
(DOCX)
