# Supplementary material for: The Olfactory Receptor OR51E1 Is Present along the Gastrointestinal Tract of Pigs, Co-Localizes with Enteroendocrine Cells and Is Modulated by Intestinal Microbiota
Source: PLoS One. 2015 Jun 15;10(6):e0129501. doi: 10.1371/journal.pone.0129501 (PMC4468170; doi:10.1371/journal.pone.0129501)
Supplement: S1 Table — Density values, n cells/mm2. (DOCX) [file pone.0129501.s001.docx]

|  | Point of measure | | | | | | | | |
| --- | --- | --- | --- | --- | --- | --- | --- | --- | --- |
| Subject | Cardia | Fundus | Pylorus | Duodenum | Jejunum | Ileum | Cecum | Colon | Rectum |
| 1 | 19 | 24.8 | 46.2 | 48 | 13.1 | 14 | 12.8 | 14.2 | 15.6 |
| 2 | 38.6 | 19.6 | 54.8 | 54.1 | 15.8 | 20.2 | 7 | 7.5 | 5.6 |
| 3 | 16.5 | 39.4 | 62.7 | 32.3 | 8.7 | 9.9 | 19.7 | 21.2 | 18.2 |
