# Supplementary material for: The Olfactory Receptor OR51E1 Is Present along the Gastrointestinal Tract of Pigs, Co-Localizes with Enteroendocrine Cells and Is Modulated by Intestinal Microbiota
Source: PLoS One. 2015 Jun 15;10(6):e0129501. doi: 10.1371/journal.pone.0129501 (PMC4468170; doi:10.1371/journal.pone.0129501)
Supplement: S2 Table — Suckling sow number, its treatment, and the final piglet age are reported for each subject. Values are per each point of sampling and in gene copies/mg RNA. (DOCX) [file pone.0129501.s002.docx]

|  |  |  |  | Gene with point of measure | | | | | |
| --- | --- | --- | --- | --- | --- | --- | --- | --- | --- |
| Subject | Sow | Age | Treatment | HMBS2_PYLORUS | HMBS2_FUNDUS | RPL4_PYLORUS | RPL4_FUNDUS | OR51E1_PYLORUS | OR51E1_FUNDUS |
| 1 | 11 | D14 | ANTB | 6503 | 10257 | 391100 | 753300 | 5872 | 4943.5 |
| 2 | 2 | D14 | CTRL | 12005 | 16937 | 1390500 | 602900 | 6951 | 4288.5 |
| 3 | 3 | D14 | CTRL | 8176 | 10485 | 847850 | 385050 | 5645 | 5228.5 |
| 4 | 12 | D14 | ANTB | 6172 | 14345 | 912300 | 1046000 | 5775.5 | 7239.5 |
| 5 | 1 | D14 | CTRL | 13210 | 8598 | 1098500 | 614700 | 6273 | 5686 |
| 6 | 8 | D14 | ANTB | 6040 | 10930 | 1094000 | 581050 | 5285 | 4815.5 |
| 7 | 4 | D14 | CTRL | 7825 | 12680 | 567000 | 696500 | 5062 | 5324.5 |
| 8 | 9 | D14 | ANTB | 7514 | 21965 | 699000 | 1024500 | 5040 | 5491.5 |
| 9 | 10 | D14 | ANTB | 7093 | 8480 | 1023000 | 639050 | 7223 | 5550.5 |
| 10 | 6 | D14 | CTRL | 7048 | 12855 | 726195 | 665000 | 5874 | 4544 |
| 11 | 5 | D14 | CTRL | 6918 | 12080 | 781750 | 416800 | 6430 | 3176 |
| 12 | 11 | D21 | ANTB | 9332 | 10940 | 676650 | 481900 | 6268.5 | 4991.5 |
| 13 | 2 | D21 | CTRL | 9308 | 11960 | 1176500 | 482100 | 5354.5 | 4789 |
| 14 | 3 | D21 | CTRL | 10855 | 4221 | 666250 | 269100 | 4197 | 3266.5 |
| 15 | 12 | D21 | ANTB | 5480 | 4684 | 614750 | 474800 | 6622 | 3262 |
| 16 | 1 | D21 | CTRL | 6758 | 9163 | 688850 | 539700 | 5447 | 5985 |
| 17 | 8 | D21 | ANTB | 6384 | 12183 | 1014500 | 619300 | 5381 | 3786 |
| 18 | 4 | D21 | CTRL | 7707 | 15730 | 1000650 | 530250 | 19030 | 4552.5 |
| 19 | 9 | D21 | ANTB | 6219 | 12505 | 667150 | 625450 | 8837.5 | 5014.5 |
| 20 | 10 | D21 | ANTB | 6117 | 14665 | 677350 | 562800 | 5146 | 3249 |
| 21 | 6 | D21 | CTRL | 4220 | 12050 | 452800 | 543550 | 4064.5 | 6589 |
| 22 | 5 | D21 | CTRL | 5628 | 11420 | 777200 | 739850 | 8936 | 4785 |
| 23 | 11 | D28 | ANTB | 11980 | 12300 | 993200 | 588550 | 4838 | 4198.5 |
| 24 | 2 | D28 | CTRL | 5616 | 8558 | 571000 | 471350 | 7949 | 9460 |
| 25 | 3 | D28 | CTRL | 6518 | 12180 | 675050 | 650550 | 6287.5 | 6655 |
| 26 | 12 | D28 | ANTB | 5857 | 8896 | 667100 | 519700 | 6598 | 3464 |
| 27 | 1 | D28 | CTRL | 5499 | 10925 | 706100 | 781850 | 9484 | 7873.5 |
| 28 | 4 | D28 | CTRL | 8149 | 7157 | 1883000 | 558400 | 7716.5 | 5506 |
| 29 | 8 | D28 | ANTB | 6892 | 7930 | 1616000 | 348750 | 5320 | 4587 |
| 30 | 9 | D28 | ANTB | 6541 | 11285 | 559950 | 703300 | 6445.5 | 6646.5 |
| 31 | 5 | D28 | CTRL | 7783 | 8615 | 675400 | 805400 | 9230 | 5844 |
| 32 | 6 | D28 | CTRL | 5942 | 7379 | 1157850 | 587850 | 4696.5 | 7965.5 |
| 33 | 10 | D28 | ANTB | 4848 | 9163 | 357000 | 592950 | 5407.5 | 6967.5 |
| 34 | 11 | D42 | ANTB | 8589 | 16300 | 985150 | 732950 | 2377 | 5818 |
| 35 | 2 | D42 | CTRL | 6916 | 7723 | 1489500 | 536800 | 4791 | 3717.5 |
| 36 | 3 | D42 | CTRL | 5959 | 8523 | 877050 | 348050 | 5482.5 | 3759.5 |
| 37 | 12 | D42 | ANTB | 5992 | 7794 | 471800 | 519850 | 5458.5 | 3511.5 |
| 38 | 1 | D42 | CTRL | 7516 | 10620 | 1011550 | 361650 | 18795 | 2523.5 |
| 39 | 8 | D42 | ANTB | 5843 | 87280 | 678850 | 580600 | 4908.5 | 3750.5 |
| 40 | 4 | D42 | CTRL | 6681 | 8820 | 522100 | 431300 | 8669.5 | 4062 |
| 41 | 9 | D42 | ANTB | 8061 | 9534 | 771000 | 484300 | 8286.5 | 4426.5 |
| 42 | 10 | D42 | ANTB | 7033 | 7264 | 911350 | 515150 | 2585.5 | 3484 |
| 43 | 6 | D42 | CTRL | 6167 | 9737 | 1738000 | 432900 | 7443.5 | 4336.5 |
| 44 | 5 | D42 | CTRL | 7611 | 7236 | 1162500 | 299600 | 5139 | 3338 |
| 51 | 17 | D14 | CTRL | 8515 | 10670 | 2666500 | 634300 | 9383.5 | 8577 |
| 52 | 20 | D14 | ANTB | 6014 | 7200 | 884600 | 429400 | 7685.5 | 6042 |
| 53 | 14 | D14 | CTRL | 7174 | 15930 | 670400 | 737500 | 8294.5 | 12365 |
| 54 | 19 | D14 | ANTB | 7090 | 11415 | 975400 | 700650 | 7637.5 | 8126 |
| 55 | 18 | D14 | CTRL | 5329 | 13380 | 324700 | 614650 | 7648 | 4776.5 |
| 56 | 22 | D14 | ANTB | 9985 | 10500 | 707750 | 933600 | 7489.5 | 6912.5 |
| 57 | 15 | D14 | CTRL | 6573 | 9580 | 713050 | 462550 | 10660 | 10237.5 |
| 58 | 16 | D14 | CTRL | 7866 | 13135 | 1008000 | 592350 | 6146.5 | 6390 |
| 59 | 13 | D14 | CTRL | 7857 | 8856 | 1165500 | 552300 | 6221.5 | 6058 |
| 60 | 23 | D14 | ANTB | 6338 | 11075 | 838450 | 502850 | 7550.5 | 4961 |
| 61 | 21 | D14 | ANTB | 8075 | 10580 | 992500 | 634300 | 6510 | 5489.5 |
| 62 | 24 | D14 | ANTB | 6396 | 9864 | 499650 | 658100 | 7520.5 | 5731.5 |
| 63 | 17 | D21 | CTRL | 4229 | 16100 | 839450 | 971800 | 5754.5 | 5621.5 |
| 64 | 20 | D21 | ANTB | 6982 | 21190 | 595200 | 956550 | 5622 | 6560 |
| 65 | 14 | D21 | CTRL | 7908 | 41440 | 980250 | 1922000 | 2946 | 7894 |
| 66 | 18 | D21 | CTRL | 6731 | 15500 | 983200 | 700150 | 7863 | 5868 |
| 67 | 19 | D21 | ANTB | 8457 | 25745 | 1437000 | 1154500 | 6483 | 4636 |
| 68 | 22 | D21 | ANTB | 8199 | 21635 | 1536333 | 1173000 | 10115 | 7138 |
| 69 | 15 | D21 | CTRL | 7114 | 12405 | 1168000 | 539400 | 8062 | 12240 |
| 70 | 16 | D21 | CTRL | 5750 | 14605 | 545600 | 629550 | 5013.5 | 4979.5 |
| 71 | 13 | D21 | CTRL | 8598 | 12320 | 1392000 | 659100 | 7375 | 8652 |
| 72 | 23 | D21 | ANTB | 7672 | 9999 | 572500 | 577650 | 5936 | 4445.5 |
| 73 | 21 | D21 | ANTB | 8103 | 15720 | 758800 | 742250 | 3210 | 7167 |
| 74 | 24 | D21 | ANTB | 6117 | 11550 | 560650 | 573450 | 7711.5 | 6371 |
| 75 | 17 | D28 | CTRL | 7239 | 18005 | 815350 | 1033000 | 12135 | 18081.5 |
| 76 | 20 | D28 | ANTB | 10228 | 10230 | 732700 | 684200 | 6639.5 | 7820.5 |
| 77 | 18 | D28 | CTRL | 5444 | 7035 | 593450 | 471200 | 7513.5 | 6397.5 |
| 78 | 22 | D28 | ANTB | 6145 | 12545 | 838500 | 1109000 | 6014 | 5093.5 |
| 79 | 15 | D28 | CTRL | 5343 | 11270 | 1152000 | 891200 | 10078.5 | 12355 |
| 80 | 16 | D28 | CTRL | 6431 | 17565 | 427500 | 819350 | 6903 | 7241.5 |
| 81 | 21 | D28 | ANTB | 14050 | 4252 | 1065000 | 344900 | 6220 | 5459.5 |
| 82 | 24 | D28 | ANTB | 5526 | 3690 | 1062500 | 426900 | 8147 | 5629 |
| 83 | 14 | D42 | CTRL | 7551 | 7201 | 621150 | 370200 | 5847 | 9500.5 |
| 84 | 20 | D42 | ANTB | 6272 | 5974 | 776950 | 335350 | 8552 | 4206.5 |
| 85 | 18 | D42 | CTRL | 7440 | 3819 | 590300 | 312400 | 15960 | 4449.5 |
| 86 | 22 | D42 | ANTB | 5512 | 7918 | 672350 | 526200 | 8543 | 8480.5 |
| 87 | 13 | D42 | CTRL | 9515 | 4911 | 624200 | 204100 | 9118.5 | 6657 |
| 88 | 16 | D42 | CTRL | 6806 | 4643 | 977650 | 267750 | 6750.5 | 4306 |
| 89 | 21 | D42 | ANTB | 4505 | 3303 | 745233 | 256100 | 4715 | 5379.5 |
| 90 | 24 | D42 | ANTB | 6833 | 4229 | 475050 | 333100 | 10975 | 4149 |
