# Supplementary material for: The Olfactory Receptor OR51E1 Is Present along the Gastrointestinal Tract of Pigs, Co-Localizes with Enteroendocrine Cells and Is Modulated by Intestinal Microbiota
Source: PLoS One. 2015 Jun 15;10(6):e0129501. doi: 10.1371/journal.pone.0129501 (PMC4468170; doi:10.1371/journal.pone.0129501)
Supplement: S3 Table — Early microbiota association treatment of the pig and intestinal loop treatment are reported for each observation. Values are in gene copies/mg RNA. (DOCX) [file pone.0129501.s003.docx]

| Microbiota | Loop treatment | Subject | OR61E1 | TBP | HMBS |
| --- | --- | --- | --- | --- | --- |
| SA | ETEC | 1 | 5479.5 | 15580 | 7405 |
| SA | ETEC | 2 | 8607.5 | 16125 | 6309.5 |
| CA | ETEC | 3 | 6897.5 | 14805 | 5044 |
| CA | ETEC | 4 | 8537 | 13645 | 3848.5 |
| SA | ETEC | 5 | 4588.5 | 19630 | 5719.5 |
| SA | ETEC | 6 | 4017 | 13330 | 6338.5 |
| CA | ETEC | 7 | 9750 | 18910 | 5235.5 |
| CA | ETEC | 8 | 15985 | 18925 | 4960.5 |
| SA | ETEC | 9 | 7952 | 15775 | 6512.5 |
| SA | ETEC | 10 | 7230 | 23720 | 7504.5 |
| CA | ETEC | 11 | 11095 | 22530 | 7378.5 |
| CA | ETEC | 12 | 9121 | 16480 | 5842.5 |
| SA | Lact.Amylovorus | 1 | 17995 | 10730 | 4096 |
| SA | Lact.Amylovorus | 2 | 13075 | 30225 | 5053 |
| CA | Lact.Amylovorus | 3 | 10405 | 16720 | 6105.5 |
| CA | Lact.Amylovorus | 4 | 11535 | 9877 | 5122 |
| SA | Lact.Amylovorus | 5 | 4784.5 | 14835 | 4821 |
| SA | Lact.Amylovorus | 6 | 8722.5 | 13590 | 5837.5 |
| CA | Lact.Amylovorus | 7 | 18060 | 13930 | 4225.5 |
| CA | Lact.Amylovorus | 8 | 17945 | 27910 | 6036 |
| SA | Lact.Amylovorus | 9 | 17320 | 30500 | 5879 |
| SA | Lact.Amylovorus | 10 | 17550 | 22695 | 6812.5 |
| CA | Lact.Amylovorus | 11 | 18760 | 13230 | 4981 |
| CA | Lact.Amylovorus | 12 | 22705 | 17085 | 5120 |
| SA | CTRL | 1 | 12615 | 16835 | 5354 |
| SA | CTRL | 2 | 13750 | 16350 | 5142 |
| CA | CTRL | 3 | 15545 | 16625 | 4996.5 |
| CA | CTRL | 4 | 10109.5 | 18615 | 5520 |
| SA | CTRL | 5 | 7325 | 16570 | 5619.5 |
| SA | CTRL | 6 | 7711 | 13065 | 5328 |
| CA | CTRL | 7 | 9042 | 9782.5 | 3394 |
| CA | CTRL | 8 | 13750 | 21625 | 4822 |
| SA | CTRL | 9 | 10570 | 14980 | 5358 |
| SA | CTRL | 10 | 15730 | 19315 | 6799.5 |
| CA | CTRL | 11 | 15125 | 20620 | 5596.5 |
| CA | CTRL | 12 | 14915 | 13870 | 4938 |
